# Prehypertension and psychosocial risk factors among university students in ASEAN countries

**DOI:** 10.1186/s12872-017-0666-3

**Published:** 2017-08-23

**Authors:** Karl Peltzer, Supa Pengpid, Vanphanom Sychareun, Alice Joan G. Ferrer, Wah Yun Low, Thang Nguyen Huu, Hla Hla Win, Erna Rochmawati, Niruwan Turnbull

**Affiliations:** 1 Department for Management of Science and Technology Development, Ton Duc Thang University, Ho Chi Minh City, Vietnam; 2 Faculty of Pharmacy, Ton Duc Thang University, Ho Chi Minh City, Vietnam; 3 ASEAN Institute for Health Development, Mahidol University, Salaya, Thailand; 4 Department of Research & Innovation, University of Limpopo, Polokwane, South Africa; 5 Faculty of Postgraduate Studies, University of Health Sciences, Ministry of Health Vientiane, Vientiane, Lao PDR; 6 Division of Social Sciences, University of the Philippines Visayas, Miagao, 5023 Iloilo, Philippines; 7 Faculty of Medicine, University of Malaya, Kuala Lumpur, Malaysia; 8 Faculty of Public Health, Hanoi Medical University, Hanoi, Vietnam; 9 Preventive and Social Medicine Department, University of Medicine 1, Yangon, Myanmar; 10 School of Nursing, Universitas Muhammadiyah Yogyakarta, Jl. Lingkar Selatan, Tamantirto, Kasihan, Bantul, DI Yogyakarta, Yogyakarta, Indonesia; 11 Faculty of Public Health, Mahasarakham University, Maha Sarakham, Thailand

## Abstract

**Background:**

Existing evidence suggests that the cardiovascular morbidities are increasing among pre-hypertensive individuals compared to normal. The aim of this study was to evaluate the prevalence of prehypertension, hypertension and to identify psychosocial risk factors for prehypertension among university students in Association of South East Asian Nation (ASEAN) countries.

**Methods:**

Based on a cross-sectional survey, the total sample included 4649 undergraduate university students (females = 65.3%; mean age 20.5, SD = 2.9, age range of 18–30 years) from 7 ASEAN countries (Indonesia, Laos, Malaysia, Myanmar, Philippines, Thailand and Vietnam). Blood pressure, anthropometric, health behaviour and psychosocial variables were measured.

**Results:**

Overall, 19.0% of the undergraduate university students across ASEAN countries had prehypertension, 6.7% hypertension and 74.2% were normotensives. There was country variation in prehypertension prevalence, ranging from 11.3% in Indonesia and 11.5% in Malaysia to above 18% in Laos, Myanmar and Thailand. In multivariate analysis, sociodemographic variables (male gender, living in an upper middle income country, and living on campus or off campus on their own), nutrition and weight variables (not being underweight and obese, having once or more times soft drinks in a day and never or rarely having chocolate or candy), heavy drinking and having depressive symptoms were associated with prehypertension.

**Conclusion:**

The study found a high prevalence of prehypertension in ASEAN university students. Several psychosocial risk factors including male gender, obesity, soft drinks consumption, heavy drinking and depression symptoms have been identified which can help in intervention programmes.

## Background

Several studies have shown an increase in the prevalence of hypertension in high and low and middle income countries [1, 2]. Similar to the trend worldwide, “hypertension is also the single most attributable cause for mortality in South-East Asia. But while in developed regions, the prevalence of hypertension appears to be stabilizing or decreasing, the rates in Southeast Asia continue to rise” [3]. If you are prehypertensive, you are more likely to become hypertensive and have a higher cardiovascular risk (compared with normotensives), and comprehensive therapeutic lifestyle modification strategy in prehypertensive subjects is indicated to reduce the risk of developing hypertension [4, 5]. In addition, studies found that hypertension and prehypertension can start in adolescence (or earlier) and continue into adulthood [6]. A study among young adults (20–30 years) found that students were at higher risk for prehypertension than the general youth population [7].

Currently, there are few studies on the assessment of prehypertension in young adults or university students in Association of Southeast Asian Nations (ASEAN). For example, in Malaysia the prevalence of prehypertension among university students was found to be 30.1% [8] to 42.9% [9], in the Philippines 13.9% [10] and in Thailand 44.5% [11]. Among Indian medical students a prehypertension prevalence rate of 45% [12] and in another study among male undergraduate students in India 55.1% [13] was found. Studies among university students in the Middle East found a prehypertension prevalence of 47.4% in Egypt [14], 39.5% in Kuwait [15], and 13.5% among females in Saudi Arabia [16].

Psychosocial risk factors associated with prehypertension or hypertension among university students and general adult population may include *sociodemographics* such as male gender [15, 17], *nutrition variables* such as Body Mass Index (BMI) overweight/obesity [7, 12, 15, 16, 18, 19], excess sodium intake [7, 20], inadequate intake of fruit and vegetables [13, 20], and *health risk behaviour variables* such as physical inactivity [20, 21], substance use, including smoking [15, 22], heavy alcohol use [23, 24] and short sleep duration [23, 25, 26]. *Psychosocial stress and support* may include depression [8, 27, 28], posttraumatic stress disorder (PTSD) [29], low life satisfaction [30], and lack of social support [25, 31, 32].

As a result, the purpose of this study was to evaluate the prevalence of prehypertension, hypertension and to identify psychosocial risk factors for prehypertension based on a cross-sectional survey of a university undergraduate population in ASEAN countries (Indonesia, Laos, Malaysia, Myanmar, Philippines, Thailand and Vietnam).

## Methods

### Study design and participants

This cross-sectional study was part of a larger investigation of a range of health behaviours in university students, and was conducted by a network of researchers in participating countries (see Acknowledgments). The university selection was a convenient sample.

### Procedure

The questionnaire utilised for data collection was developed in English, then translated and back-translated into the languages (Bahasa, Khmer, Lao, Myanmar, Thai, Vietnamese) of the participating countries. Research assistants administered the questionnaire at the end of a teaching class (inclusion criteria: all students present in class). In each study country, undergraduate students were surveyed in classrooms selected through as stratified random sample procedure (one university department randomly selected from each faculty as a primary sampling unit, and for each selected department randomly ordered undergraduate courses). Participation rates were in all countries more than 90%, except for Indonesia 86% and Myanmar 73%.

### Measures

#### Blood pressure (BP) measurements and classification

Three consecutive measurements of systolic and diastolic BP were measured by well-trained research assistants using appropriately sized cuff and the bell of a standard stethoscope, with at least 1 min between assessments after the participant had rested for 5 min in a sitting position. Average blood pressure was calculated arithmetically for the 3 measurements of each systolic and diastolic blood pressure. Missing values were excluded from being ncluded in the study. Blood pressure classification was done using JNC 7 algorithm [33]. Prehypertension was defined as systolic blood pressure (SBP) measurement of 120–139 mmHg or diastolic blood pressure (DBP) of 80–89 mmHg in people who were not taking antihypertensive medication. Hypertension was defined as SBP ≥140 mmHg and/or DBP ≥90 mmHg and/or current use of antihypertensive medication. Normotension was defined as BP values <120/80 mmHg in students who were not taking antihypertensive medication [34]. All respondents were initially asked if they have ever been diagnosed with hypertension and if they did, whether or not they have been taking any kind of drugs or other treatment for the last 2 weeks and last 12 months.

*Socio-demographic factor* questions included age, gender, residential status, subjective socioeconomic background, and country income status [35].

#### Nutrition variables

*Anthropometric measurements. Height* (without footwear) using a stadiometer and *weight* (without footwear and any heavy accessories) using a calibrated weighing scale was measured. Body mass index (BMI) was calculated as weight in kg divided by height in metre squared. Body mass index (BMI) was classified according to Asian criteria: normal weight (18.5 to <23.0), overweight (23.0 to <25.0) and 25+ as obese [36].

*Fruit and vegetable (FV) consumption* was assessed with two questions, “How many servings of fruit do you eat on a typical day?” and “How many servings of vegetables do you eat on a typical day?” (One standard serving = 80 g) [37]. Cronbach alpha for this fruit and vegetable measure was 0.74. Insufficient fruit and vegetable consumption was defined as less than five servings of fruits and/or vegetables a day [37]. Cronbach alpha for the two questions in this sample was FV 0.68.

Additional dietary variables included: (a) trying to avoid eating foods that contain fat and cholesterol (yes, no); (b) adding salt to meals (1 = usually to 4 = never); (c) eat a meal that includes meat (beef, pork, lamb, etc.) (1 = at least once a day to 5 = never); [38], (d) times per day usually drink carbonated soft drinks in the past 30 days (1 = None to 7 = 5 or more times per day, (e) Number of days ate food from a fast food restaurant in the past 7 days (1 = 0 days to 8 = 7 days) [39], and (f) frequency of consuming chocolate or candy (1 = more than once a day to 6 = never).

#### Health behaviour

*Physical activity*. Physical activity was assessed using the self-administered International Physical Activity Questionnaire (IPAQ) short version, for the last 7 days (IPAQ-S7S). We used the instructions given in the IPAQ manual [40], and categorized physical activity (short form) according to the official IPAQ scoring protocol [41] as low, moderate and high.

*Tobacco use* was assessed with the question: Do you currently use one or more of the following tobacco products (cigarettes, snuff, chewing tobacco, cigars, etc.)? Response options were “yes” or “no” [42].

Past month *binge drinking* was assessed with one item of the Alcohol Use Disorder Identification Test [43].

*Sleep duration*. The survey also included one question about self-reported hours of sleep, on average in a 24 h period. The category of 7–8 h of sleep was used as reference. This reference category was chosen because some studies reported that those who slept 7 or 8 h usually had the lowest mortality risk [44].

#### Psychosocial stress measures

*Post traumatic stress disorder (PTSD).* Breslau’s 7-item screener was used to identify PTSD symptoms in the past month; participants who scored four or more were considered to have a positive screen for PTSD [45]. (Cronbach alpha = 0.77).

The *Centres for Epidemiologic Studies Depression Scale (CES-D:* 10 items) was used to assess depressive symptoms, and scores 15 or more were classified as severe depressive symptoms [46] (Cronbach’s alpha = 0.71).

#### Well-being and social support

*Life satisfaction* was assessed with one item, “All things considered, how satisfied are you with your life as a whole?” Response option ranged from 1 = very satisfied to 5 = very dissatisfied [38].

*Social support* was measured with three items from the Social Support Questionnaire [47]. (Cronbach’s alpha 0.64).

### Data analysis

The data were analyzed using IBM-SPSS for Windows, version 23 (Chicago, Illinois, USA). Descriptive statistics were used to calculate frequency of study variables of the study population and Chi-square test to assess difference in proportions. Logistic regression analyses were used to test significant determinants of prehypertension status, with prehypertension serving as the dichotomous outcome variable (prehypertension versus normotensives) and sociodemographics, health, nutrition and psychosocial stress as the independent predictor variables.

## Results

### Sample characteristics

The total sample included 4649 undergraduate university students (females = 65.3%; mean age 20.5, SD = 2.9, age range of 18–30 years) from 7 ASEAN countries (Indonesia, Laos, Malaysia, Myanmar, Philippines, Thailand and Vietnam). Among countries, the sample size ranged from 231 in Indonesia to 1019 in Malaysia. Overall, 19.0% of the undergraduate university students across ASEAN countries had prehypertension, 6.7% hypertension and 74.2% were normotensives. Of those who were classified as having hypertension, 29.8% were currently using of antihypertensive medication. There was country variation in prehypertension prevalence, ranging from 11.3% in Indonesia and 11.5% in Malaysia to above 18% in Laos, Myanmar and Thailand. The hypertension prevalence ranged from 3 to 4% in Laos, Malaysia and Philippines to above 12% in Indonesia and Thailand. Overall, prehypertension and hypertension prevalence was higher among male than female university students (see Tables 1 and 2).

**Table 1:** Sample characteristics (*N* = 4649)

Sociodemographics	Normotensives	Prehypertension	Hypertension	*P*-value
*All*	3451 (74.2)	885 (19.0)	313 (6.7)	
*Age in years*				
18–19	1193 (74.1)	200 (11.7)	117 (7.3)	0.448
20–21	1294 (74.1)	330 (18.9)	123 (7.0)	
22–30	964 (74.6)	255 (19.7)	73 (6.7)	
Gender				
Female	2453 (80.8)	422 (13.9)	161 (5.3)	<0.001
Male	997 (61.8)	463 (28.7)	152 (9.4)	
Economic family background				
Wealthy	1055 (74.4)	256 (18.1)	107 (7.5)	0.218
Poor	2396 (74.2)	629 (19.5)	206 (6.4)	
Country income				
Upper middle income	1189 (65.4)	446 (24.5)	184 (10.1)	<0.001
Lower middle income	2262 (79.9)	439 (15,5)	129 (4.6)	
Country				
Indonesia (*N* = 231)	177 (76.6)	16 (11.3)	28 (12.1)	<0.001
Laos (*N* = 717)	550 (76.7)	139 (19.4)	28 (3.9)	
Malaysia (*N* = 1019)	751 (73.7)	36 (11.5)	10 (3.2)	
Myanmar (*N* = 314)	268 (85.4)	190 (18.6)	78 (7.7)	
Philippines (*N* = 775)	638 (82.3)	112 (14.5)	25 (3.2)	
Thailand (*N* = 800)	438 (54.8)	256 (32.8)	106 (13.3)	
Vietnam (*N* = 793)	629 (79.3)	126 (15.9)	38 (4.8)	
Living arrangement				
Lives with parents/guardians	1017 (79.3)	206 (16.1)	53 (4.6)	<0.001
Live away from parents	2431 (72.3)	679 (20.2)	254 (7.6)

**Table 2:** Sample characteristics (*N* = 4649)

Nutrition variables	Normotensives	Prehypertension	Hypertension	*P*-value
Body Mass Index (BMI)				
Normal	1922 (75.6)	470 (18.5)	152 (6.0)	<0.001
Underweight	810 (81.1)	126 (12.6)	62 (6.2)	
Overweight	330 (71.3)	101 (21.8)	32 (6.9)	
Obese	307 (56.5)	171 (31.5)	65 (12.0)	
Usually eat salt	1175 (77.4)	252 (16.6)	91 (6.0)	0.002
Avoid foods containing fat and cholesterol	1241 (73.5)	316 (18.7)	11 (7.8)	0.102
Insufficient fruits and vegetables	2636 (75.2)	651 (18.6)	220 (6.3)	0.046
Soft drinks (once or more a week)^1^	682 (67.7)	232 (23.0)	94 (9.3)	0.003
Fast food (once or more a week)^1^	1240 (73.2)	338 (20.0)	115 (6.8)	0.006
Red meat at least once a day	1980 (75.0)	503 (19.0)	158 (6.0)	0.065
Chocolate or candy^a^				
Never or rarely	926 (67.3)	311 (22.6)	139 (10.1)	<0.001
1–6 times a week	973 (75.0)	238 (18.3)	87 (6.7)	
Once or more times a day	364 (75.4)	85 (17.6)	34 (7.0)	
Health behaviour				
Physical activity				
Low	1974 (75.1)	473 (18.0)	182 (6.9)	0.069
Moderate	1462 (73.3)	409 (20.5)	124 (6.2)	
High	469 (69.8)	160 (23.8)	43 (6.4)	
Current tobacco use	114 (65.9)	47 (27.2)	12 (6.9)	0.018
Heavy drinking at least monthly	199 (63.4)	95 (30.3)	20 (6.4)	<0.001
Sleep duration				
7–8 h	1572 (75.5)	370 (17.8)	140 (6.7)	0.385
6 or less hours	1574 (73.2)	429 (20.0)	147 (6.8)	
9 or more hours	294 (75.6)	73 (18.8)	22 (5.7)	
Psychosocial stress				
Depression (severe)	308 (64.3)	114 (23.8)	57 (11.9)	<0.001
Posttraumatic stress disorder (PTSD)	812 (72.4)	230 (20.5)	79 (7.0)	0.312
Well-being and social support				
Life satisfaction				
Low	1918 (71.9)	545 (20.4)	204 (7.6)	<0.001
Medium	456 (71.3)	102 (20.4)	41 (8.2)	
High	1174 (79.4)	237 (16.0)	68 (4.6)	
Social support				
Low	1565 (71.6)	450 (20.6)	172 (7.9)	<0.001
High	1875 (76.6)	433 (17.7)	141 (5.8)	

^a^Not assessed in Laos and Philippines

### Association between psychosocial risk factors and prehypertension

In bivariate analyses, sociodemographic variables (male gender, living in an upper middle income country, and living on campus or off campus on their own), nutrition and weight variables (not being underweight and obese, not usually adding salt in food, having once or more times soft drinks in a day and never or rarely having chocolate or candy), health behaviour variables (high physical activity, current tobacco use and heavy drinking), having depressive symptoms, low life satisfaction and low social support were associated with prehypertension.

In multivariate analysis, sociodemographic variables (male gender, living in an upper middle income country, and living on campus or off campus on their own), nutrition and weight variables (not being underweight and obese, having once or more times soft drinks in a day and never or rarely having chocolate or candy), heavy drinking and having depressive symptoms were associated with prehypertension (see Table 3).

**Table 3:** Pedictors of pre-hypertension compared to normal blood pressure

Sociodemographics	UOR (95% CI)	AOR (95% CI)^a^
*Age in years*		
18–19	1 (Reference)	---
20–21	1.01 (0.85–1.21)	
22–30	1.05 (0.87–1.27)	
Gender		
Female	1 (Reference)	1 (Reference)
Male	2.70 (2.32–3.14)***	2.56 (2.17–3.02)***
Economic family background		
Wealthy	1 (Reference)	---
Poor	0.92 (0.79–1.09)	
Country income		
Upper middle income	1 (Reference)	1 (Reference)
Lower middle income	0.52 (0.45–0.60)***	0.52 (0.42–0.64)***
Living arrangement		
Lives with parents/guardians	1 (Reference)	1 (Reference)
Lives away from parents/guardians	1.38 (1.16–1.64)***	1.39 (1.03–1.89)*
Nutrition variables		
Body Mass Index (BMI)		
Normal	1 (Reference)	1 (Reference)
Underweight	0.64 (0.51–0.79)***	0.67 (0.52–0.87)**
Overweight	1.25 (0.98–1.60)	1.11 (0.86–1.43)
Obese	2.28 (1.84–2.82)***	1.94 (1.55–2.43)***
Usually eat salt	0.77 (0.65–0.91)**	0.97 (0.82–1.16)
Avoid foods containing fat and cholesterol	1.00 (0.86–1.17)	---
Insufficient fruits and vegetables	0.88 (0.73–1.07)	---
Soft drinks (once or more a week)^1^	1.34 (1.11–1.61)**	1.56 (1.27–1.92)***
Fast food (once or more a week)^1^	0.95 (0.79–1.13)	---
Red meat at least once a day	1.00 (0.86–1.16)	---
Chocolate or candy^1^		
Never or rarely	1 (Reference)	1 (Reference)
1–6 times a week	0.73 (0.60–0.88)**	0.74 (0.60–0.90)**
Once or more times a day	0.70 (0.53–0.91)**	0.82 (0.62–1.09)
Health behaviour		
Physical activity		
Low	1 (Reference)	1 (Reference)
Moderate	1.05 (0.88–1.24)	0.89 (0.74–1.07)
High	1.36 (1.08–1.71)**	1.25 (0.98–1.59)
Current tobacco use	1.65 (1.16–1.64)**	1.03 (0.70–1.51)
Heavy drinking at least monthly	1.96 (1.32–2.54)***	1.91 (1.43–2.54)***
Sleep duration		
7–8 h	1 (Reference)	---
6 or less hours	0.86 (0.74–1.01)	
9 or more hours	0.91 (0.69–1.20)	
Psychosocial stress and support		
Depression (severe)	1.51 (1.20–1.90)***	1.32 (1.00–1.76)*
Posttraumatic stress disorder (PTSD)	1.14 (0.96–1.35)	---
Life satisfaction		
Low	1 (Reference)	1 (Reference)
Medium	1.01 (0.79–1.28)	1.01 (0.78–1.31)
High	0.71 (0.60–0.84)***	0.97 (0.78–1.21)
Social support		
Low	1 (Reference)	1 (Reference)
High	0.80 (0.69–0.93)**	0.90 (0.77–1.05)

^a^Hosmer & Lemeshow Chi-square = 15.14, *P* = 0.057; Nagelkerke R^2^: 0.12; ***P < .001; **P < .01; *P < .05

*UOR* Unadjusted Odds Ratio; *AOR* Adjusted Odds Ratio; *CI* Confidence Interval; ^1^not assessed in Laos and Philippines

## Discussion

The study found among a large sample of undergraduate university students across seven ASEAN countries a prehypertension prevalence of 19.0%, which seems generally lower than previous in the region [8, 9, 11–13] and in the middle east [14, 15]. There was some country variation in prehypertension prevalence, ranging from 11.3% in Indonesia and 11.5% in Malaysia to above 18% in Laos, Myanmar and Thailand. Students residing in upper middle income countries such as Thailand had a higher prevalence of prehypertension than those in lower middle income countries. These country differences in terms of prehypertension prevalence may be attributed to different stages of the epidemiologic transition of the participating student populations in the various countries. As found in some previous studies [15, 17], this study found a significant higher prevalence of prehypertension among male (28.7%) than female (13.9%) students. Nevertheless, a large proportion of, especially male, university students in ASEAN countries seem to suffer from prehypertension requiring comprehens ive lifestyle modification programmes [4].

Compared with students who were residing with their parents or guardians, students who were living on campus or off campus on their own were at a higher risk for prehypertension. It is possible that students living away from their parents are more influenced by their peers in terms of lifestyle changes increasing the development of prehypertension. Regarding weight variables, being underweight was protective and being obese increased the odds of having prehypertension. The association between overweight/obesity and prehypertension and hypertension has been confirmed in a number of studies [7, 12, 15, 16, 18, 19]. Further, having once or more times soft drinks in a day increased the odds of prehypertension and moderate consumption of chocolate or candy (1–6 times a week) was protective of prehypertension. In a systematic review, Ried et al. [48] found that “flavanol-rich chocolate and cocoa products may have a small but statistically significant effect in lowering blood pressure by 2-3 mm Hg in the short term.” Unlike a few previous studies [7, 13, 20], this study did not find an association between excess sodium intake, inadequate intake of fruit and vegetables and prehypertension. A previous study [49], found an association between sodium intake and hypertension in the older age group but not in the younger age group, so it is possible that other risk factors (such as obesity) for raised blood pressure play a larger role than sodium intake at the age of young adulthood [50]. Another study among Chinese adults also did not find an association between salt intake and prhypertension [51]. Another possible reason for this result could be recall bias resulting from self-reporting of salt intake [51].

Regarding health risk behaviours, this study found that heavy drinking students had an higher odds of having prehypertension, as also found in previous studies [23, 24]. Although current evidence suggests that the moderate consumption of alcohol lowers the blood pressure, chronic ethanol consumption (≥ three drinks a day) is associated with an increased incidence of hypertension [52]. Tobacco use was found in bivariate analysis associated with prehypertension prevalence, as also some previous studies found a significant association [15, 22]. Unlike several previous studies [20–23, 25, 26], this study did not find an association between physical inactivity, short sleep duration and prehypertension. However, several other studies also did not find a clear association between physical inactivity and raised blood pressure [53, 54]. It is possible that the driving force behind increased blood pressure in this study population is not solely physical inactivity but is linked to other factors such as obesity, which still can be advocated for in order to improve a healthy body [53]. Further, in a previous review [55], it was found that the association between short sleep and higher blood pressure and hypertension was stronger among middle aged adults, which could possibly mean that among a younger age (emerging adulthood) effects of short sleep on raised blood pressure are not yet found.

In terms of psychosocial stress and support, this study found in agreement with previous studies [8, 27, 28] that depression was positively related to prehypertension. “There is considerable evidence suggesting that hyperreactivity of the sympathetic nervous system and genetic influences are the underlying mechanisms in the relationship between depression and hypertension.” [56] Further, in bivariate analysis this study found and association between low life satisfaction, and lack of social support and prehypertension, as found in previous studies [25, 30–32].

### Study limitations

First, the sample was representative of the population from which it was drawn as all participants were participants of undergraduate students of one university. However, for the same reason, the sample might not be representative of universities. Apart from anthropometric and blood pressure measurements, a limitation of the study was that all the other information collected in the study was based on self reporting. It is possible that certain behaviours were under reported. Further, it was a cross-sectional study and the temporal or antecedent consequent relationships between risk factors and the development of prehypertension cannot be established in such studies. Longitudinal study of this cohort will enable analysis of the impact of such behaviour in early life on subsequent development of health problems.

## Conclusion

The study found high prevalence, in particular among men, of prehypertension in a large sample of university students across seven ASEAN countries. Often, in university students, prehypertension may not be discovered until late. This is because university students are generally healthy and will normally not go for a routine health check-up. The data from this study draws attention to the importance of examining the blood pressure of young persons. Several psychosocial risk factors including male gender, obesity, soft drinks consumption, heavy drinking and depression symptoms have been identified which can help in intervention programmes.

## Abbreviations

ASEAN: Association of Southeast Asian Nations
BMI: Body Mass Index
FV: Fruit and vegetable
IPAQ: International Physical Activity Questionnaire (IPAQ)
PTSD: Posttraumatic Stress Disorder
